# Lower hippocampal volume partly mediates the association between rs6859 in the *NECTIN2* gene and Alzheimer’s disease: new findings from causal mediation analysis of ADNI data

**DOI:** 10.3389/fnagi.2026.1715773

**Published:** 2026-02-11

**Authors:** Aravind Lathika Rajendrakumar, Konstantin G. Arbeev, Olivia Bagley, Anatoliy I. Yashin, Svetlana Ukraintseva

**Affiliations:** 1 Biodemography of Aging Research Unit, Social Science Research Institute, Duke University, Durham, NC, United States; 2 Institute for Health Equity Research, Icahn School of Medicine at Mount Sinai, New York, NY, United States

**Keywords:** Alzheimer’s disease, causal mediation analysis, hippocampal volume, infections, *NECTIN2*, rs6859

## Abstract

**Introduction:**

Alzheimer’s disease (AD) is a complex disorder influenced by many factors. The rs6859 polymorphism in the *NECTIN2* gene has been consistently linked to AD risk. The *NECTIN2* is involved in vulnerability to infections, which could contribute to neurodegeneration. We hypothesized that hippocampal volume (HV), a biomarker of neurodegeneration, may mediate the connection between the *NECTIN2* polymorphism and AD.

**Methods:**

The analysis was conducted using data from the Alzheimer’s Disease Neuroimaging Initiative (ADNI). Linear mixed models were used to evaluate the association between SNP rs6859 and normalized hippocampal volumes over time. Multivariable linear and logistic regression models were used to estimate the associations between SNP rs6859 and median hippocampal volumes, and between SNP rs6859 and median hippocampal volumes and AD, adjusting for potential confounders. Causal mediation analyses (CMA) were performed using previously fitted logistic and linear models to estimate the mediating role of hippocampal volumes in the association between rs6859 and AD.

**Results:**

We found that smaller HV significantly mediates the association between rs6859 in *NECTIN2* and AD risk. Carrying the rs6859 risk allele (A) was associated with lower right HV (*β* = −0.16, *p* = 0.03), left HV (*β* = −0.14, *p* = 0.04), and total HV (*β* = −0.15, *p* = 0.04) in linear mixed models. These associations were significant only in males. The mediated effects for the right and left HV were 42.75 and 49.76%, respectively.

**Discussion:**

Our results indicate that hippocampal atrophy may mediate the association between NECTIN2 polymorphism and AD risk, although the borderline significance of these associations warrants confirmation in other populations.

## 1 Introduction

Alzheimer’s disease (AD) is a major cause of neurodegeneration and cognitive impairment in older adults. Progressive atrophy of brain structures occurs in AD due to neurodegenerative changes that worsen with the severity of the disease (Deture and Dickson, 2019). The hippocampus is the part of the brain responsible for memory formation (Van Der Flier and Scheltens, 2009). Hippocampal atrophy is both a key indicator of neurodegeneration and an important biomarker of AD pathology (Rao et al., 2022). Pathological features of AD typically first manifest in and around this region (Knierim, 2015). Hippocampal volume (HV) measured by magnetic resonance imaging (MRI) may detect pathological changes that are useful for predicting dementia, even in the absence of clinical symptoms (Achterberg et al., 2019).

AD is a multifactorial disorder arising from the interplay of various factors, including genetic variation and infections, among others (Yashin et al., 2018; Armstrong, 2019; Flowers and Rebeck, 2020; Montagne et al., 2020; Akushevich et al., 2023; Ukraintseva et al., 2023a, 2023b, 2024b). An increasing body of research suggests that infections may play an important role in AD, dementia, and neurodegeneration (Urosevic and Martins, 2008; Porcellini et al., 2010; Itzhaki, 2016; Shinjyo and Kita, 2021; Cairns et al., 2022; Ukraintseva et al., 2023b, 2024a, 2024b; Popov et al., 2024; Rajendrakumar et al., 2025). Seemingly mild infections, such as urinary tract infections (UTIs), can profoundly elevate inflammation levels (Howe et al., 2012), and disrupt hippocampal nerve plasticity (Darwish et al., 2022). These pathological changes are largely irreversible, despite treatment with appropriate medications (Darwish et al., 2022). Infections can also impair hippocampal metabolism (Zorzo et al., 2023). It is not yet clear though whether the infections contribute to AD directly, by inducing neurodegeneration, or if they are indicators of vulnerability to AD caused by other factors. Most of the evidence linking infections to AD risk comes from observational studies and may be subject to bias. A causal mediation analysis (CMA) that leverages genetic risk factors involved in both AD and infections may provide better understanding of the role of neurodegeneration in the associations between these factors and AD development.

Genetic variation in the *NECTIN2* gene is a plausible candidate for such analysis. It has been associated with both AD and vulnerability to infections, especially to herpes viruses (Logue et al., 2011; Yashin et al., 2018). The *NECTIN2* protein participates in the maintenance of cellular tight junctions and neurons (Molfetta et al., 2019; Ogawa et al., 2022). Hence, a variation in this gene could potentially influence the spread of pathogens in the brain (Duara and Barker, 2022; Ogawa et al., 2022). A single-nucleotide polymorphism (SNP) rs6859 in *NECTIN2* is one of the strongest AD risk factors identified in the genome-wide association study (GWAS) (Yashin et al., 2018). It has also been associated with cognitive changes, phosphorylated tau, pneumonia, and the protective effects of pneumonia and flu vaccination against AD and AD risk (Ukraintseva et al., 2023a, 2023b; Rajendrakumar et al., 2024b, 2024a). We recently found that prior infections and the rs6859 risk allele (A) are associated with reduced hippocampal volume in the UK Biobank participants (Ukraintseva et al., 2024a). This indicates a possibility that hippocampal atrophy might be one of the mechanisms underlying the association between the rs6859 (A) allele and increased AD risk.

In this study, we explored whether the rs6859 (A) allele was associated with trajectories of hippocampal volume in the ADNI dataset. We further performed causal mediation analysis to evaluate whether reduced hippocampal volume mediates the association between rs6859 (A) allele in *NECTIN2* and AD risk.

## 2 Materials and methods

### 2.1 Study population, hippocampal volume measurement, and genetic data extraction

Data used in the preparation of this article were obtained from the Alzheimer’s Disease Neuroimaging Initiative (ADNI) database.^1^ The ADNI was launched in 2003 as a public-private partnership, led by Principal Investigator Michael W. Weiner. The primary goal of ADNI has been to test whether serial magnetic resonance imaging (MRI), positron emission tomography (PET), other biological markers, and clinical and neuropsychological assessment can be combined to measure the progression of mild cognitive impairment (MCI) and early AD. We conducted a secondary data analysis, and no participants were enrolled directly in our study. In ADNI, consenting participants are enrolled through a staggered recruitment approach and have varying follow-up times. Medical history, vital signs, and other clinical parameters were collected at screening. Neuroimages and biomarkers were measured in a subset of participants based on a standard protocol developed by clinical imaging experts (Jack et al., 2008). Hippocampal volumetric data in ADNI were acquired using 1.5 Tesla (T) and 3 Tesla (T) MRI scanners from selected manufacturers and were automatically extracted using FreeSurfer software (Fischl, 2012; Jack et al., 2008; Hartig et al., 2014). Genotyping in the ADNI was conducted using different genotyping arrays: Human610-Quad BeadChip, Illumina HumanOmniExpress BeadChip, and Illumina Infinium Global Screening Array v2 (GSA2), and the data were stored in the Plink format.

#### 2.1.1 Data linkage

We linked the hippocampal volume data with demographics, SNP rs6859, and clinical variables, including diabetes medication use by their participant roster ID (RID) (see text footnote 1). Diabetes medication use (Yes/No) was identified from prescription data by matching drug names to the Anatomical Therapeutic Chemical (ATC) classification system.^2^ We relabeled smoking and alcohol history as Ever/Never and extracted SNP rs6859 alleles using the—recode command in Plink 1.90 beta (Purcell and Chang, n.d.). These were further verified with the summary SNP information file to ensure accurate data linkage.

### 2.2 Statistical analysis

All statistical analyses were performed using R software version 4.3.2 (R Core Team, 2021). Analyses were limited to individuals with complete data for all covariates. We included age, hippocampal volumes, diabetes (yes/no), SNP rs6859, smoking (ever/never), alcohol use (ever/never), visits, duration of education, race, and married status (ever/never) in the regression models. Continuous variables were summarized as means ± standard deviation, and categorical variables were represented as frequencies and percentages. Univariate and multivariate visualizations were generated with the *ggplot2* package (Wickham, 2016). Histograms of hippocampal volume and its longitudinal trajectories by age and clinical visits, stratified by rs6859 allele status, are presented in Supplementary material. We applied an ordered quantile normalization (ORQ) transformation (Peterson and Cavanaugh, 2019) using the *bestNormalize* package to normalize hippocampal volumes (Peterson, 2023). This kind of transformation is suitable for improving the fit of parametric models. Variable collinearity was assessed prior to modeling. First, linear mixed models were fit using the *lme4* package to assess the association between SNP rs6859 and normalized hippocampal volumes over time (Bates et al., 2015). Additionally, total hippocampal volume (the sum of the right and left hippocampal volumes) was considered an outcome.

Allele dosages of SNP rs6859 were included in the linear and logistic regression models for the mediator and outcome, assuming an additive genetic model. Age, sex, marital status, number of visits, education duration, diabetes, race, smoking, and alcohol use were controlled in the analysis. The “*dredge*” function in the *MuMin* package was used for variable selection (Barton, 2022). It allows reproducible, automated model selection by ensuring that all possible combinations of model terms are considered to determine the parsimonious model based on the Akaike Information Criterion (AIC). A random intercept was specified for different starting values of hippocampal volumes and random slopes for differences in clinical visits across individuals. The data were apportioned accordingly for sex-stratified analysis, and associations were computed within each group. Statistical associations in the regression models were considered significant for two-sided *p*-values less than 0.05.

We next estimated the parameters from the causal mediation analyses (CMA) using median values from all readings for hippocampal volumes and other continuous covariates generated by the *dplyr* package (Wickham et al., 2022). Given the extreme values in the predictors, a more stable measure could be obtained by using average readings rather than the raw values (Rajendrakumar et al., 2023). CMA mimics different scenarios by varying the exposure relationship with the outcome, conditional on the mediator values likely at different levels of exposure to influence the potential outcomes (Rijnhart et al., 2021b). As we have a continuous mediator and a binary outcome (AD), the CMA method can be used to reliably decompose the exposure effects into natural direct and indirect effects (Rijnhart et al., 2020). To investigate if the *NECTIN2* gene polymorphism influences AD risk through hippocampal volume reduction, we conducted a covariate-adjusted mediation analysis using the *Medflex* package (Steen et al., 2017). We used the *RNomni* package to transform the summarized hippocampal measures (McCaw, 2020). The main advantages of *RNomni* are that it applies the Rank-based inverse normal transformation (INT), which is particularly useful for small sample sizes, and that variability in the model is ensured when estimating associations. Only those predictors of AD selected by the algorithm were carried forward to the CMA for covariate adjustment. We used the “neWeight” function in the same package to apply the ratio-of-mediator-probability weighting (RMPW) method to create a pseudo dataset for counterfactual estimation, using inverse probability weighting (Lange et al., 2012; Steen et al., 2017). At first, the expected value of the mediator at observed and counterfactual levels of exposure was computed. These estimated values were used by the *neWeight* function to create an in-built pseudo-dataset by reweighting on the expected mediator value computed earlier. In this way, subject-specific weights are calculated, which are subsequently accounted for in a regression that models the exposure-mediator-outcome relationship and finally provides separate coefficients for the Natural Direct Effect (NDE) and the Natural Indirect Effect (NIE) (Steen et al., 2017; Rijnhart et al., 2021a). Due to the logit scale, we have also interpreted the NDE and NIE as odds ratios for better understanding. We computed the proportion of mediated effects (PE) by dividing NIE by Total effects (TE), since the package lacks a built-in function for this calculation.

## 3 Results

### 3.1 Sample characteristics

**Figure 1:**
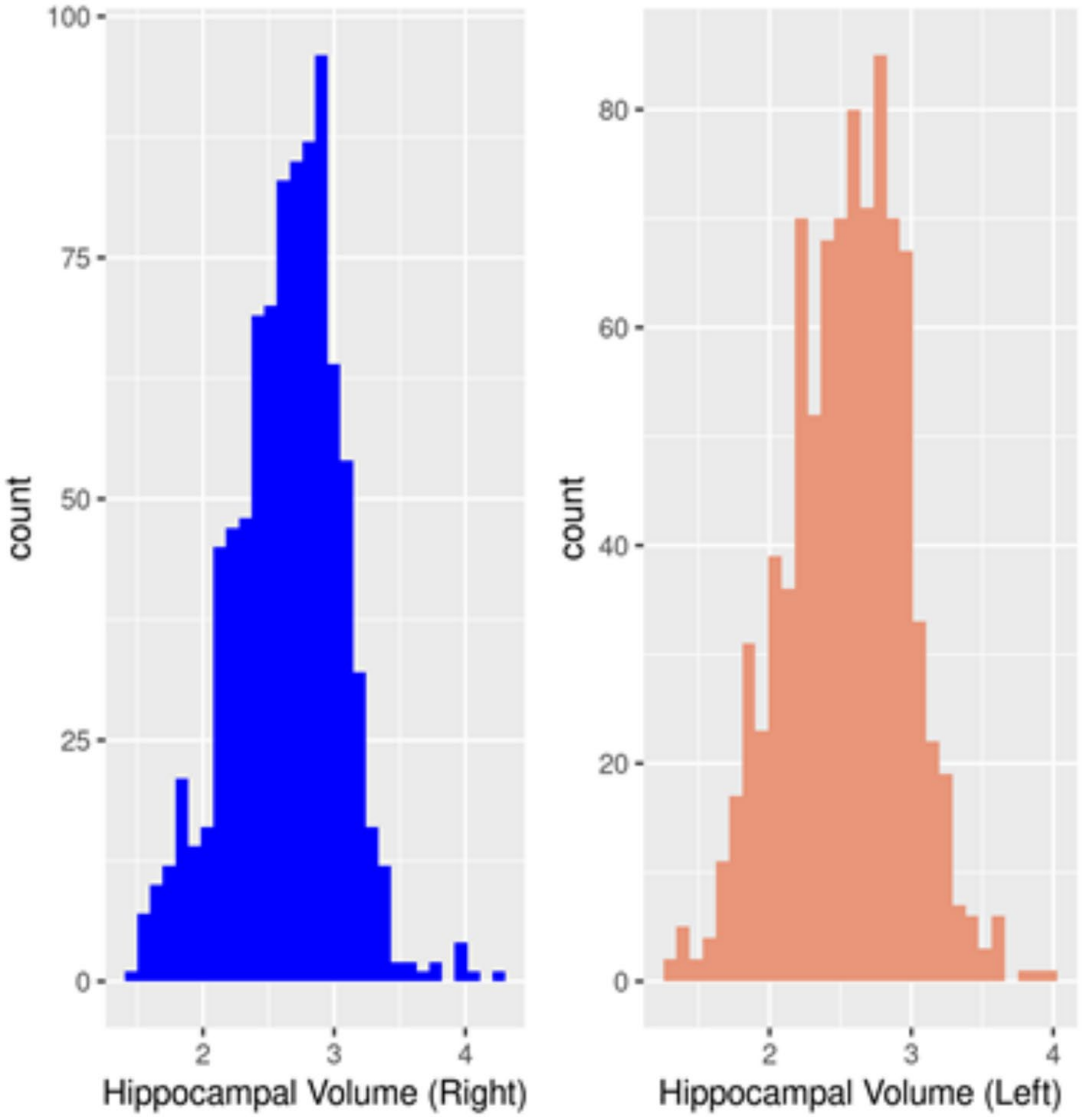
Histogram of hippocampal volume.

**Figure 2:**
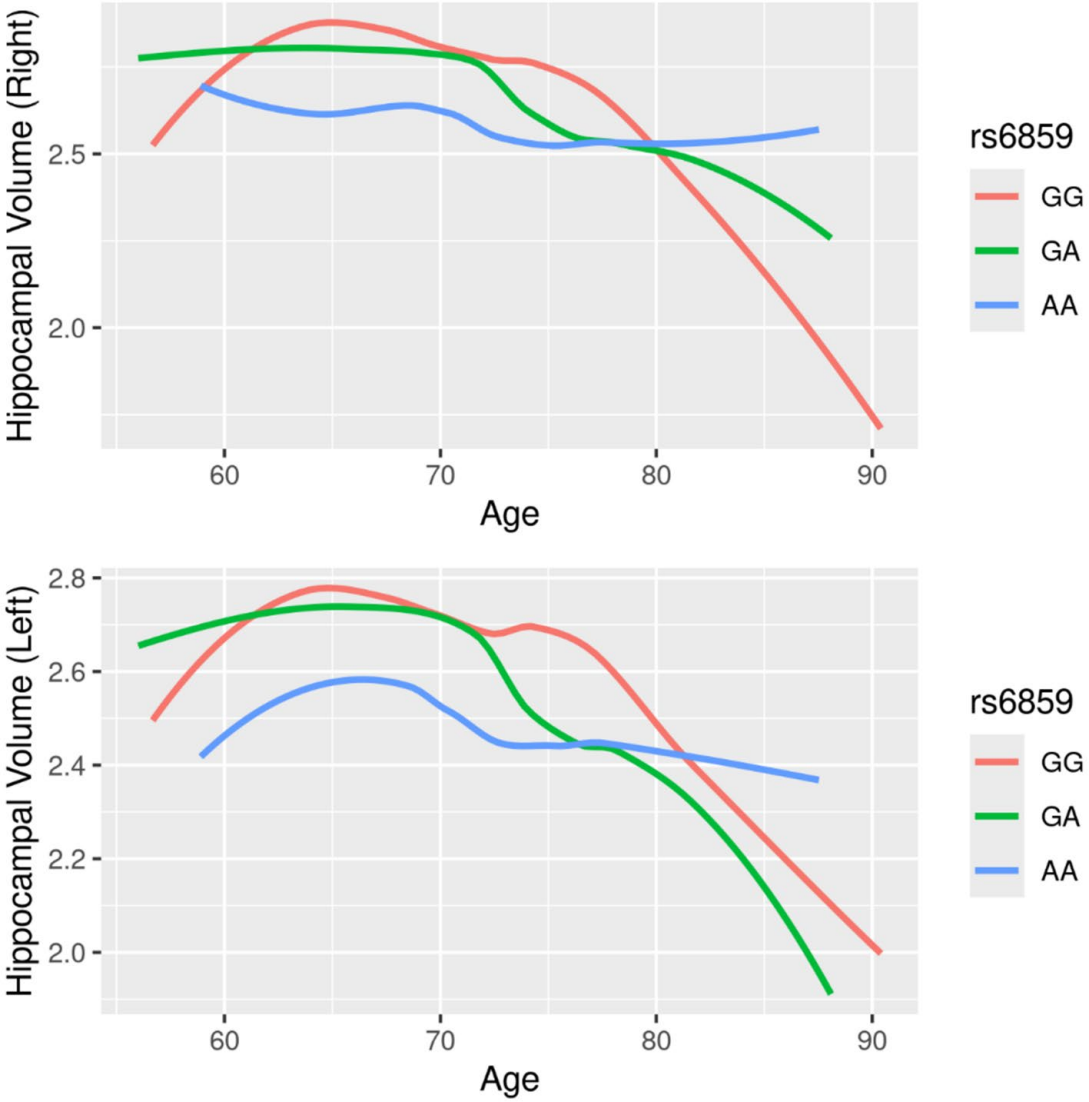
Smoothed trajectories of hippocampal volume with age, stratified by rs6859 allele status.

**Figure 3:**
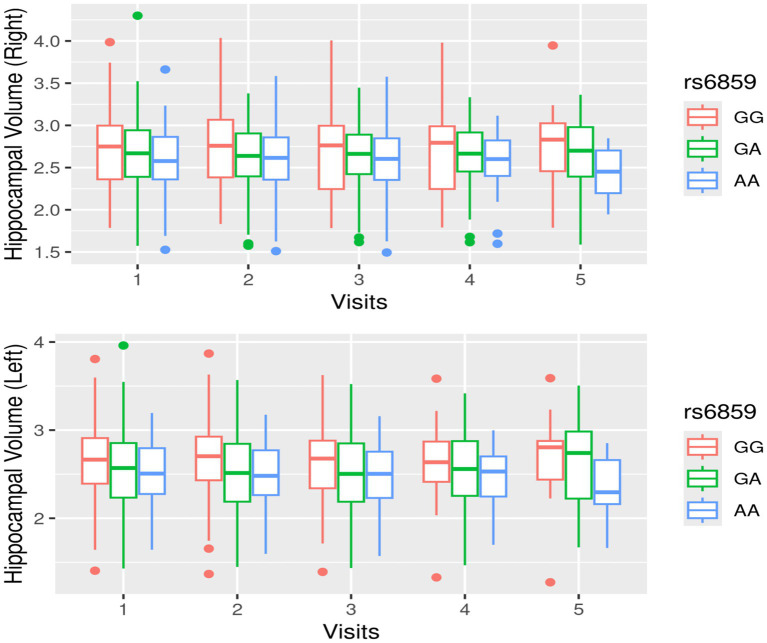
Longitudinal change in hippocampal volume with clinical visits, stratified by rs6859 allele status.

A total of 902 records from to 318 participants were analyzed, as detailed in the flowchart (Supplementary Figure S1). The histograms in Figure 1 show that the right hippocampal volume had a slightly leptokurtic distribution in comparison to the left hippocampal volume. There were differences in the trajectories of hippocampal volumes with age when stratified by rs6859 allele status, as shown in Figure 2. For the younger age range, the hippocampal volume trajectories remained stable. Among participants, those with the GG genotype showed a rapid decline in right hippocampal volume with age. With aging, participants in the GA group experienced a faster decline in LHV than those in the GG group, showing an opposite trend to that observed in RHV. The change in hippocampal volume for the AA genotype was not pronounced in either hippocampal region, and the relative difference between age groups was minimal. Longitudinal changes in hippocampal volume across clinical visits, stratified by rs6859 allele status, are shown in Figure 3. Carriers of the rs6859 A risk allele exhibited decreased hippocampal volumes with an increasing number of clinical visits.

### 3.2 Predictors of right hippocampal volume and differences by sex

Table 1 shows the coefficients for the variables associated with longitudinal changes in right hippocampal volume. Increased age and the number of clinical visits were associated with lower hippocampal volume (*p* < 0.001). Being female has a greater effect on right hippocampal volume reduction than having diabetes. Carriage of A alleles of rs6859 predicted a lower right hippocampal volume than non-carriers (−0.155, *p* = 0.033). Marital status, although included in the model, did not achieve statistical significance. AIC-based regression identified a similar set of variables for RHV in both sexes (Supplementary Tables S1, S2). Notably, the variable selection methods chose age and behavioral risk factors, such as alcoholism, which were not identified for LHV or in the subgroups. However, their influences differed, showing more prominence in males. None of the variables, excluding age and sex, were predictive in females. Across both sexes, SNP rs6859 was not significant, although the estimate was larger and approached the significance threshold (*p* = 0.06) in males. Similarly, as seen in the case of LHV, males with diabetes were more likely to have a lower RHV.

**Table 1:** Linear mixed model estimates of rs6859 and covariates with right hippocampal volume (*n* = 318, observations = 902).

Variables	Estimates	95% CI	*p*
Age	−0.062	−0.074, −0.050	0.000***
Diabetes (yes)	−0.376	−0.637, −0.115	0.005**
Married Status (never)	0.365	−0.069, 0.799	0.102
rs6859	−0.155	−0.297, −0.013	0.033*
Sex (female)	−0.630	−0.832, −0.427	0.000***
Visits	−0.022	−0.033, −0.012	0.000***

Estimates adjusted for education, race, smoking, alcohol, and diabetes. Abbreviations: CI, Confidence Interval; p, *p*-value. *p < 0.05; **p < 0.01; ***p < 0.001.

### 3.3 Predictors of left hippocampal volume and differences by sex

Table 2 presents the results of the LMM analysis for the left hippocampal volume. In the whole dataset, an increase in the dosage of the rs6859 A allele was associated with a reduction in left hippocampal volume over time (*β* = −0.139, *p* = 0.044). Females had an increased risk of hippocampal volume loss (*β* = −0.584, *p* < 0.001). Increasing age, clinical visits, and a diagnosis of diabetes were also identified as risk factors influencing left hippocampal atrophy. No significant influence of education or marital status on the evolution of left hippocampal volume was observed. Supplementary Tables S3, S4 display the results of the sex-stratified analysis. All variables included in the final model for males were statistically significant and adversely affected LHV.

**Table 2:** Linear mixed model estimates of rs6859 and covariates with left hippocampal volume (*n* = 318, observations = 902).

Variables	Estimates	95% CI	*p*
Age	−0.058	−0.070, −0.047	0.000***
Diabetes (yes)	−0.284	−0.539, −0.044	0.025*
Education	0.032	−0.005, 0.071	0.093
Married Status (never)	0.360	−0.079, 0.748	0.08
rs6859	−0.139	−0.269, 0.003	0.044*
Sex (female)	−0.584	−0.782, −0.383	0.000***
Visits	−0.025	−0.0343, −0.015	0.000***

Estimates adjusted for race, smoking, and alcohol. CI, Confidence Interval; p, *p*-value. *p < 0.05; **p < 0.01; ***p < 0.001.

Most importantly, SNP rs6859 was selected (−0.242, *p* = 0.014) only in this category. In contrast, a different set of variables was chosen in females, except for age and clinical visits, which had the same direction of effect as in males. Higher education and never being married were associated with improved LHV, with the former demonstrating a borderline significance (*p* = 0.05).

### 3.4 Predictors of total hippocampal volume and differences by sex

Table 3 details the variables associated with total hippocampal volume. The results showed a similar pattern to that estimated for LHV. Compared to LHV, the mixed model estimate for SNP rs6859 on THV was marginally higher (−0.145, 95% CI: −0.285, −0.004, *p* = 0.043) but lower than RHV. Age, sex, diabetes, and clinical visits were strongly associated with total hippocampal volume loss. Education and marital status variables were retained but were non-significant in the final model.

**Table 3:** Linear mixed model estimates of rs6859 and covariates for THV (*n* = 318, observations = 902).

Variables	Estimates	95% CI	*p*
Age	−0.065	−0.077, −0.054	0.000***
Education	0.031	−0.007, 0.070	0.120
Diabetes (Yes)	−0.334	−0.589, −0.078	0.01*
rs6859	−0.145	−0.285, −0.004	0.043*
Sex (female)	−0.607	−0.813, −0.401	0.000***
Visits	−0.022	−0.031, −0.012	0.000***
Married status (never)	0.375	−0.050, 0.801	0.087

Estimates adjusted for race, smoking, and alcohol. Abbreviations: CI, Confidence Interval; p, *p*-value. ^*^p < 0.05; ^**^p < 0.01; ^***^p < 0.001.

Regarding the sex-stratified analysis, more variables were included for males (Supplementary Tables S5, S6). SNP rs6859, diabetes, age, and clinical visits were associated with THV in males. The smoking and alcohol history was also chosen among the predictors but was not significant. The estimated coefficient of THV showed that the only common variables between males and females were age and visits.

### 3.5 Participant characteristics in the summarized mediation analysis

Table 4 gives the characteristics of participants included in the dataset for the mediation analysis. Most participants were older than 60 years, male, better educated, and white. Few individuals had up to five clinical visits. Almost 22% reported having smoked during their lifetime. However, only a few participants reported ever consuming alcohol, which is unusual and may indicate a potential reporting problem. More than a quarter of the participants were diagnosed with AD, and about 18% had diabetes. Regarding hippocampal volume, the right hippocampus was slightly higher than the left. A large proportion of participants (~74%) carried the rs6859 risk allele (A), and about half carried the *APOE4* risk allele.

**Table 4:** Participant characteristics in the mediation analysis sample (*n* = 318).

Variables	Median (%)	IQR	Range
Age, years	73.0	68.1–78.0	56.4–89.6
Sex, Male, n (%)	165 (51.9%)		
Education, years	16.0	14.0–18.0	8.0–20.0
Visits	3	2–4	1–5
RHV	2.6	2.3–2.9	1.5–4.3
LHV	2.5	2.2–2.8	1.3–3.9
THV	5.2	4.6–5.7	3.0–8.2
Race			
White	291 (91.5%)		
Other	27 (8.5%)		
Smoking			
Ever	69 (21.7%)		
Alcohol			
Ever	7 (2.2%)		
AD (Yes)	85 (26.7%)		
Diabetes (Yes)	57 (17.9%)		
SNP rs6859^$$^			
GG	84 (26.4%)		
GA	159 (50.0%)		
AA	75 (23.6%)		
*APOE4*			
0	163 (51.25%)		
1	121 (38.15%)		
2	34 (10.79%)		

‘$$’ indicates genotype frequencies, and IQR refers to the interquartile range.

### 3.6 Causal mediation analysis

Here, we quantified the direct and indirect effects of the A allele at rs6859 on hippocampal volumes, separately and jointly, to gather evidence for a causal relationship. For this, we used only covariates deemed significant for AD and hippocampal volumes. The resulting estimates were interpreted as conditional probabilities of the mediator, given the rs6859 and confounder levels in the model. First, we computed estimates for the right hippocampal volume (Table 5), suggesting no direct effect of rs6859 on it (*β* = 0.220, *p* = 0.229). In contrast, we observed a mediating role for the RHV on the AD risk predicted by SNP rs6859 (*β* = 0.165, *p* = 0.033), implying a proportion of mediated effects (PME) of 42.75%. As expected, aging individuals were more susceptible to AD. Intriguingly, the number of visits was protective against AD risk in the model.

**Table 5:** Confounder-adjusted mediation analysis showing the direct and indirect effects of rs6859 on AD through the right hippocampal volume.

Variables	Estimates	95% CI	*p*
rs6859 (direct)	0.220	−0.146, 0.573	0.229
rs6859 (indirect)	0.165	0.010, 0.314	0.033*
Age	0.042	0.002, 0.079	0.029*
Married status (never)	−2.102	−11.032, 17.137	0.769
Visits	−0.340	−0.520, −0.148	0.000***
TE	0.386	−0.005, 0.757	0.047*

Variables included for adjustment were selected based on significant predictors of AD from the full model, which comprised rs6859, age, smoking status, sex, diabetes status, race, marital status, alcohol consumption, education level, and clinical visits. TE, Total Effect. *p < 0.05; **p < 0.01; ***p < 0.001.

In Table 6, we present the conditional probability estimates for the AD-rs6859 relationship, with left hippocampal volume change as a mediating variable. Once again, the direct effect for rs6859 was not statistically significant (*p* = 0.259). We observed that the NIE observed was higher than that seen for the RHV, as reflected by a 4.80% increased risk. Adjusting for the covariate effects, LHV reduction was associated with a 22.7% higher risk for AD. Furthermore, the change in LHV with rs6859 risk alleles accounted for a greater proportion of the mediated effects, reaching 49.76%. A unit increase in age was associated with a 4.08% higher risk of AD. Contrary to expectations, a higher number of clinical visits was related to with negative AD risk.

**Table 6:** Confounder-adjusted mediation analysis showing the direct and indirect effects of rs6859 on AD through the left hippocampal volume.

Variables	Estimates	95% CI	*p*
rs6859 (direct)	0.206	−0.158, 0.559	0.259
rs6859 (indirect)	0.205	0.039, 0.363	0.012*
Age (years)	0.041	0.001, 0.078	0.035*
Married status (never)	−2.175	−11.078, 16.995	0.761
Visits	−0.341	−0.521, −0.150	0.000***
TE	0.412	0.015, 0.789	0.036*

Variables included for adjustment were selected based on significant predictors of AD from the full model, which comprised rs6859, age, smoking status, sex, diabetes status, race, marital status, alcohol consumption, education level, and clinical visits. TE, Total Effect. ^*^p < 0.05; ^**^p < 0.01; ^***^p < 0.001.

Details of the causal mediation estimates for the direct and indirect effects of rs6859 on AD through total hippocampal volume (THV) are presented in Table 7. In the causal mediation analysis (CMA), there was no evidence for a direct effect of rs6859 on the outcome (estimate = 0.207, 95% CI: −0.157 to 0.558, *p* = 0.255). However, the indirect effect of rs6859 was statistically significant (estimate = 0.190, 95% CI: 0.024 to 0.349, *p* = 0.021), indicating a 22.1% increased risk per additional A allele, acting exclusively through hippocampal volume loss.

**Table 7:** Confounder-adjusted mediation analysis showing the direct and indirect effects of rs6859 on AD through the total hippocampal volume.

Variables	Estimates	95% CI	*p*
rs6859 (direct)	0.207	−0.157, 0.558	0.255
rs6859 (indirect)	0.190	0.024, 0.349	0.021*
Age (years)	0.042	0.002, 0.079	0.031*
Married status (never)	−2.131	−11.05, 17.085	0.766
Visits	−0.340	−0.521, −0.149	0.000***
TE	0.398	0.003, 0.772	0.042*

Variables included for adjustment were selected based on significant predictors of AD from the full model, which comprised rs6859, age, smoking status, sex, diabetes status, race, marital status, alcohol consumption, education level, and clinical visits. TE, Total Effect. ^*^p < 0.05; ^**^p < 0.01; ^***^p < 0.001.

Furthermore, the total effect (TE) was statistically significant, confirming that THV partially mediates the impact of rs6859 on AD. The computed proportion of mediated effect was 47.7%, suggesting that nearly half of the total effect is explained via the indirect pathway. Additionally, covariate effects for THV remained consistent with those observed in other CMA analyses.

## 4 Discussion

Our study found that the rs6859 (A) allele, a risk factor for AD in the *NECTIN2* gene, which is involved in vulnerability to infections, is associated with reduced hippocampal volume in ADNI participants. This supports our recent finding in the UK Biobank (Ukraintseva et al., 2024a). The CMA revealed that a lower hippocampal volume may account for a substantial portion (almost half) of the detrimental effect of rs6859 (A) on AD risk. One potential explanation for this effect is that *NECTIN2* is a key component of adherens junctions, playing a role in cell–cell adhesion and mediating viral entry into the brain. Hence, its variation may affect the brain’s permeability and vulnerability to infections. This hypothesis needs further confirmation.

Another notable finding is that the impact of the rs6859 polymorphism differed across hippocampal spheres, which is likely, as certain hippocampal regions are more susceptible than others in AD (Greene and Killiany, 2012). The left and right hippocampi serve different cognitive functions; the left with verbal memory and the right with spatial memory (Burgess et al., 2002). Our results did not align with a study that concluded there was no region-specific hippocampal loss in AD (Lan et al., 2024). However, the authors still found greater left-sided hippocampal atrophy in semantic dementia cases. On the other hand, Lindberg and colleagues analysed the hippocampal shape across multiple dementia subtypes and observed a consistent left hippocampal predominance in volume loss (Lindberg et al., 2012). Indeed, this aligns with our data, which includes participants with varying levels of cognition and those with AD.

In terms of statistical significance, a much-attenuated effect on right hippocampal volume was observed in males. Our findings contrast with previous evidence from the UK Biobank. There, the results indicated that female carriers of the A allele of rs6859 with prior infections faced a risk of hippocampal loss, whereas males did not (Ukraintseva et al., 2024a). Since, to our knowledge, no detailed studies have explored the gender-specific effects of *NECTIN2* on the brain or AD, we are unable to speculate on the underlying reasons. Our findings are novel but require replication in other cohorts.

AD is sometimes called type 3 diabetes due to certain commonalities with type 2 diabetes (T2D) pathology (Nguyen et al., 2020). Regardless, variable selection for the AD outcome did not support its inclusion in the final model. Published studies have often found conflicting results regarding the causal nature of the T2D-AD associations. Recently, more reliable evidence emerged from a large GWAS study that applied Mendelian randomization (MR) analysis, which reported no direct connection between AD and T2D (Liu et al., 2024). That said, indirect pathways may still exist. For instance, our previous research using ADNI data demonstrated that diabetes negatively affects brain regions associated with AD (Rajendrakumar et al., 2025). Diabetes was strongly associated with hippocampal volume loss in our LMM models. We attribute this finding to the reported role of diabetes in abnormal hippocampal activation, which impairs learning and memory (Huang et al., 2016). Another plausible pathway is that diabetes can adversely affect hippocampal structure by disrupting neurogenesis and neuroplasticity (Ho et al., 2013).

The CMA primarily aims to identify a causal link between the A allele of rs6859 and AD counterfactually while treating visits as a covariate, which addresses a different research question altogether. Counterfactual mediation models are considered more capable than other mediation models, as they estimate real-world, interpretable effects, require fewer assumptions, and can be used to model exposure-mediator interaction effects. The smallest mediated effect was observed for the right hippocampal volume (RHV) at 42.75%, while the largest was found for the left hippocampal volume (LHV) at 49.76%. For the THV, the estimated effect was closer to that of the LHV, suggesting that the *NECTIN2* gene has a stronger impact on the left hippocampus and that the THV effect is largely due to this.

There are several possible mechanisms by which genetic variations in *NECTIN2* may affect hippocampal volume. *NECTIN2* is a relatively understudied gene, primarily because of its location near the well-studied *APOE4* and *TOMM40* locus (Kulminski et al., 2020; Mizutani et al., 2022; Ukraintseva et al., 2023b). The SNP rs6859 is located in the non-coding region of the *NECTIN2* gene, and its polymorphisms may interfere with the miRNA binding, leading to neurological damage (Llorens et al., 2017; Pathak et al., 2020; Li et al., 2024). We checked the Human Protein Atlas to understand the impact of *NECTIN2*. While *NECTIN2* mRNA is present in the hippocampus, its expression has not been detected in either glial or neuronal cells, suggesting a potential post-transcriptional regulation or expression in other cell types (The Human Protein Atlas, 2025).

The *NECTIN2* gene is highly pleiotropic and influences multiple phenotypes, including a causal effect on LDL-C independent of the *APOE* effect (van der Graaf et al., 2020). Elevations in LDL-C and other lipids have been shown to increase the AD pathologic features markedly and are inversely correlated with hippocampal volume (Wingo et al., 2022; Kang et al., 2023). The association between higher LDL-C and hippocampal volume was partially mediated by Aβ aggregation, underlining a complex web of pathological effects (Kang et al., 2023). Additionally, these *NECTIN2*-related changes could affect astrocyte and neuronal health, which, in turn, influence the hippocampal mass (Miyata et al., 2016; Lana et al., 2021). As previously described, *NECTIN2* polymorphism may also influence pTau levels (Rajendrakumar et al., 2024a). The pTau, as a mediator, explained only a limited portion of the rs6859 association with AD, suggesting the impact of other mediators, including neurodegeneration. Our previous research supported associations among *NECTIN2* and infections and hippocampal volume (Yashin et al., 2018; Ukraintseva et al., 2023a, 2023b; Rajendrakumar et al., 2025). One should note, however, that while these prior findings are broadly in line with the causal relationship observed in this study, mechanistic link remains suggestive and requires confirmation in further research involving larger sample sizes.

As regards the strengths of our study, the *medflex* estimate is robust with respect to outcome prevalence and non-collapsibility issues frequently encountered in the CMA of binary outcomes, due to the use of counterfactual-based statistics (Samoilenko and Lefebvre, 2021). The robustness arises from counterfactual estimations being calculated by estimating causal effects through simulations of varying exposure-mediator relationships, which is quite distinct from computing conditional effects in traditional regression models. We also report significant improvement in understanding how the *NECTIN2* gene may influence AD risk through its endophenotypes, greatly increasing explained variance to 69.16% from 19.40% (Rajendrakumar et al., 2024a). Furthermore, we demonstrated statistically significant associations using longitudinal measures and summary data, systematically adjusting for established confounders.

Regarding the limitations, we were mainly constrained by sample size, as hippocampal measurements were collected for only a subset of participants in the cohort. This led to further reductions during data linkage. In our mixed models, we specified hippocampal volume differences between individuals as a random effect to account for inter-individual variability. However, the non-inclusion of total intracranial volume (TIV), which varies across individuals, may have affected the accuracy of the estimates. Our mixed-model analysis suggested an inverse relationship between frequent clinical visits and hippocampal atrophy, whereas in CMA, visits showed a protective association with AD. The primary reason for this discrepancy is that individuals in the linked dataset had already been diagnosed with AD at the time of recruitment, which likely masked any expected associations with study visits. The majority of our samples were White, which may substantially limit the generalizability of our findings to other racial and ethnic groups.

Furthermore, because the dataset predominantly consists of older individuals, it may be necessary to examine these associations in younger populations. For these reasons, reduced statistical power may have influenced the results, leading to borderline significant *p*-values. While our findings suggest that hippocampal atrophy substantially mediates the association between rs6859 and AD risk, these results should be interpreted with caution. Further validation in independent populations is needed before drawing definitive conclusions. Beyond infections, weakened immunity related to aging and genetics can further contribute to accelerated neurodegeneration (Ukraintseva et al., 2024b). Studying inflammation in relation to the *NECTIN2* gene and AD may help explain part of the remaining unexplained pathway.

## 5 Conclusion

Our study suggests that hippocampal atrophy can significantly mediate the association between rs6859 (A) in the *NECTIN2* gene and AD risk. Depending on the hippocampal region involved, this mechanism could account for nearly half the risk associated with rs6859 (A). Given the borderline significance of these associations, replication in independent populations is warranted.

## Data Availability

This study used de-identified human data provided by the Alzheimer’s Disease Neuroimaging Initiative (ADNI) (https://adni.loni.usc.edu/about/). The ADNI data are hosted on the Image and Data Archive (IDA) by the Laboratory of Neuro Imaging (LONI) at the University of Southern California. This data is not freely available to the public but can be accessed after applying for the data access through the LONI IDA and receiving an approval by the ADNI Data Sharing and Publications Committee. Specific policies governing this process can be found online at https://adni.loni.usc.edu/data-samples/adni-data/.

## Footnotes

1
http://adni.loni.usc.edu

2
https://www.who.int/tools/atc-ddd-toolkit/atc-classification

## Group member of the Alzheimer’s Disease Neuroimaging Initiative

Data used in preparation of this article were obtained from the Alzheimer’s Disease Neuroimaging Initiative (ADNI) database (adni.loni.usc.edu). As such, the investigators within the ADNI contributed to the design and implementation of ADNI and/or provided data but did not participate in analysis or writing of this report. A complete listing of ADNI investigators can be found at: http://adni.loni.usc.edu/wp-content/uploads/how_to_apply/ADNI_Acknowledgement_List.pdf.
